# Content validity of digital health competence measures for nursing in South Africa

**DOI:** 10.3389/fpubh.2026.1874126

**Published:** 2026-09-07

**Authors:** Amanda Cromhout, Felicity Daniels, Jennifer Chipps

**Affiliations:** Faculty of Community and Heath Sciences, School of Nursing, University of the Western Cape, Cape Town, South Africa

**Keywords:** content validity, DigiComInf, DigiHealthCom, digital competence, nurses, South Africa

## Abstract

**Introduction:**

The increasing use of digital technology to improve healthcare outcomes for patients and institutions requires that nurses have adequate levels of digital competence, measured by psychometrically sound measures. However, most measures are developed in high-income countries and have not been adapted to other settings. Therefore, this study aimed to determine the content validity of two measures, developed in Finland, that measure digital competence (*DigiHealthCom*) and influences on digital competence (*DigiComInf*), for their application in South Africa.

**Methods:**

Participants were predominantly nurses from private and public facilities in South Africa who completed an online content validity rating scale (*n* = 12) South Africa by rating the relevance and clarity of each item and subscale of competence (*DigiHealthCom*) and influence (*DigiComInf*). Of these, ten participated in an online focus group discussion. Ratings were analysed by calculating individual item content validity indices (I-CVI) and scale and subscale average content validity indices (S-CVI/Ave).

**Results:**

I-CVI values for relevance and clarity were mostly >.78, except for some items in the “human-centred remote counselling competence” subscale (*DigiHealthCom*) and one item in *DigiComInf*. S-CVIs were sufficient for *DigiHealthCom* (S-CVI_relevance_/Ave = 0.86, S-CVI_clarity_/Ave = 0.87) and *DigiComInf* (S-CVI_relevance_/Ave = 0.95, S-CVI_clarity_/Ave = 0.89). The focus group discussion highlighted several concerns, such as the absence of discerning, contextually relevant definitions for the constructs measured, lack of clarity about the target population, and applicability of certain items to different healthcare contexts.

**Discussion:**

Adaptations included adding relevant definitions, clarifying the target population or management level, adding a “not applicable” option to the rating scale and clarifying the meaning or context using appropriate alternative words. The final measure was approved by the participants and the original authors of the measures.

## 1 Introduction

The development and application of digital health technology has changed the way in which health care is delivered. Benefits include faster and safer delivery of care (1), workload management, and improved clinical decision-making and efficiency (2), thus optimising outcomes for patients and institutions (1, 2). Digital competence is defined as “the skills and practices required to use new technologies in a meaningful way and as a tool for learning, working and leisure time, understanding the essential phenomena of digital technologies in society as well as in one’s own life, and the motivation to participate in the digital world as an active and responsible actor” (3). Several factors influence the acquisition of digital competence. Jarva et al. (4) reported that attitudes towards and experience in using digital solutions, receiving support from colleagues, the influence of the manager, and the availability of continuous development and orientation were associated with digital health competence in healthcare professionals. Erfani et al. (5) found that higher levels of digital health competence were associated with higher levels of education, having more professional experience, working in a hospital setting, and increased use of digital solutions at work or during free time.

To enable the effective use of digital health technology, health professionals – especially nurses as the biggest health care professional group – should have high levels of digital competence to effectively engage with these technologies. Evidence from local research shows poor levels of informatics skills in nurses and have identified the need for prioritising these skills in continuing education (6). Similarly, there have been recommendations to increase the integration of nursing informatics in the undergraduate nursing curriculum to properly prepare students for a digital health care environment (7). The need for digital health competence requires valid, reliable and contextually relevant measures of digital health competence and related aspects. Two measures were developed in Finland to determine digital health competence (*DigiHealthCom*) and the factors that influence digital health competence (*DigiComInf*) in health professionals (8). *DigiHealthCom* comprises 42 items that measure the digital health competence of healthcare professionals using five different subscales: (1) human-centred remote counselling competence (16 items), (2) digital solutions as part of work (9 items), (3) information and communication technology (ICT) competence (5 items), (4) competence in utilising and evaluating digital solutions (8 items) and (5) the ethical competence related to digital solutions (4 items). *DigiComInf* comprises 15 items that measure factors related to educational and organisational factors associated with digital health competence using three different subscales: (1) support from management (6 items), (2) organisational practices as part of digital competence development (4 items), and (3) colleagues’ adoption and influence (5 items) (8). These measures differ from other measures in that *DigiHealthCom* measures more dimensions of digital health competence, which is useful to determine specific training needs to enhance digital health competence; and *DigiComInf* evaluates the organisational and educational factors associated with digital health competence, and helps to understand the facilitators and barriers to the development of digital health competence (8).

However, most measures, such as *DigiHealthCom* and *DigiComInf*, were developed in high-income countries and may not be contextually relevant in other settings. For use in South Africa, it was important to assess whether these measures were valid for local use. Content validity is an important component of validity and is defined as the extent to which the content is relevant to and representative of the target construct for a particular assessment purpose (9, 10). Inferences from measures without satisfactory content validity may be suspect, even if other indicators of validity are satisfactory (11). Therefore, the aim of this study was to determine the content validity of *DigiHealthCom* and *DigiComInf* for nurses – as the predominant group of healthcare professionals globally and in South Africa – as part of the South African leg of an international collaboration with the University of Oulu in Finland.

## 2 Materials and methods

A content validity study was conducted, which included an online content validity rating scale and an online content validity focus group. A total of 20 healthcare professionals from South Africa, predominantly from nursing, were invited to participate in the study. The healthcare professionals had to hold a position related to digital health and digitisation (whether from education, public practice, or private practice) with at least 20 years’ experience in their respective roles. Sample sizes between six to 10 participants were deemed adequate (10, 12, 25).

### 2.1 Instruments

#### 2.1.1 Content validity rating scale

The content validity rating scale contained the items from the *DigiHealthCom* and *DigiComInf* and a rating scale for relevance and clarity. Participants were asked to rate each subscale and item in terms of relevance (R1 = *Not relevant*; R2 = *Item needs revision*; R3 = *Relevant but minor revision*; R4 = *Very relevant*) and clarity (C1 = *Not clear*; C2 = *Item needs revision*; C3 = *Clear but minor revision*; C4 = *Clear*) in the South African context. Additionally, participants could include comments about their ratings.

### 2.2 Procedure

A pilot study was conducted during the development phase of *DigiHealthCom* and *DigiComInf* to assess the feasibility and face validity of the English measures, and to obtain feedback on aspects such as readability, completion time, and clarity of the measures (8). Our study thus focused on the content validity of these scales for the South African healthcare setting. The procedure included two parts: expert ratings of a content validity rating scale, created on REDCap, containing the English version of *DigiHealthCom* and *DigiComInf*; and a single online focus group, which was conducted in English. Prior to the focus group, the link to the content validity rating scale was sent to the participants and participants were invited to the focus group. An information sheet on the purpose of the study, what would be expected of participants, and all relevant ethical aspects such as informed consent, voluntary participation, audio recording of the session and confidentiality accompanied the invitation to participate. The online focus group was facilitated by the primary author. The facilitator confirmed the purpose of the study and explained the background of the scales; that the facilitator will read each item of *DigiHealthCom* and *DigiComInf* after which the relevant subscales and items of the measures would be rated, commented on, and discussed in terms of their relevance and clarity in the South African context. The other two facilitators ensured that participant responses were captured accurately using a google docs document that was shared with all participants prior to and during the focus group. This enabled participants to confirm if their responses were captured correctly. Participants who did not complete the content validity rating scale prior to the focus group could complete it during the focus group prior to each discussion. The focus group was planned to last 3 hours, with time allocated per subscale per measure for the rating and discussion of the items and subscales. Despite the time allocation, we allowed flexibility in time to ensure unhindered participant input. Participants were allowed a refreshment break halfway through the focus group. The focus group did not exceed the 3 hours planned. The focus group was recorded and a summary transcription provided. No coding was done as responses were specific to each item. The comments and suggestions for more contextual wording of the items were incorporated to produce the amended measures. The amended versions, which were sent to the participants for their approval, were approved by all participants. In addition, the amended versions were approved by the original authors of the measures.

### 2.3 Data analysis

Content validity was determined by calculating individual item scores (I-CVI) and the total average scores (S-CVI/Ave) (13). The I-CVI was calculated as the number of experts who rated an item as R3 (*Relevant but minor revision*) or R4 (*Very relevant*) for relevance or C3 (*Clear but minor revision*) or C4 (*Clear*) for clarity, divided by the number of experts. Where items were not rated by all experts, the denominator was adjusted and the I-CVI value was calculated based on the number of experts who did rate the item. I-CVI values should be equal to one when there are five or fewer reviewers—the standards can be relaxed when there are more reviewers (6–10), with values ≥0.78 generally considered acceptable (12). Items not meeting the cut-off criteria suggest areas for revision, deletion, or substitution. The total average score (S-CVI/Ave) was calculated as the average of the I-CVIs across the measure. S-CVI/Ave values ≥0.90 are acceptable (10, 14) for content validity, with values of 0.80 as the lower limit for acceptability (13, 15).

### 2.4 Ethics statement

After ethics approval was obtained from the Biomedical Research Ethics Committee (BMREC) of the University of the Western Cape (BM25/2/7), participants were invited to participate in the study. Participants were informed about the purpose of the study and that they were expected to complete and discuss their ratings on the content validity rating scale where appropriate. All participants gave written informed consent, which included consent to be recorded. Participation was voluntary.

## 3 Results

### 3.1 Participants

Twelve (12) participants completed the content validity rating scales. Participant roles included two (2) nursing informatics specialists in education, two (2) nurse educators (primary care and midwifery); a nurse informaticist in government public health services (*n* = 1); two (2) private health nursing services digital experts, a private nurse informaticist consultant (*n* = 1); a nurse manager in primary care (1); two nurses government public health services (*n* = 2) and nurse education (*n* = 1). Ten (10) participants (male = 2, female = 8) participated in the focus group of which two were non-nurse healthcare professionals (education in psychology: *n* = 1; education in physiotherapy: *n* = 1). As the focus was on the digital health work environment, the contributions of the two non-nurse healthcare professionals were included in the study. Participants were from two provinces in South Africa (Western Cape = 8, Gauteng = 2), which was viewed as representative as nursing education is regulated across South Africa with the same competencies regardless of whether they work in public or private facilities. In addition, as the focus was on the digital health work environment, the contributions of the two non-nurse healthcare professionals were included in the study.

### 3.2 *DigiHealthCom* content validity ratings for relevance and clarity and participant recommendations

#### 3.2.1 Overall ratings for the *DigiHealthCom*

The S-CVI values of the total measure were both acceptable (S-CVI_relevance_/Ave = 0.86; S-CVI_clarity_/Ave = 0.87). Recommended changes across the overall measure included changing “digital solutions” to “digital tools” to ensure conceptual clarity; changing “customer” to “client” to fit the South African context; adding a “not applicable” option to the response scale; and amending the administration instructions accordingly. Supplementary Table S1 shows the comparison between the original and amended items of *DigiHealthCom*, and Supplementary Table S2 shows the final version of *DigiHealthCom* suggested for use in South Africa.

#### 3.2.2 Subscale 1: human-centred remote counselling competence

The S-CVI values for this subscale were acceptable (S-CVI_relevance_/Ave = 0.80; S-CVI_clarity_/Ave = 0.80), although lower than for the other subscales (Table 1). For most items the I-CVI_relevance_ were ≥0.82 except for items 6, 10, 11, 12, 16 that were below the ≥0.78 cut-off (Table 1). I-CVI_clarity_ were ≥0.82 except for items 1, 6, 11, 12, and 16 (Table 1).

**Table 1:** Ratings and CVI values for DigiHealthCom.

Item	# Experts	Relevance		Clarity	
		R3/R4	I-CVI	C3/C4	I-CVI
Human-centred remote counselling competence					
1. I can act in reciprocal (aiming towards respect and equality) interaction with the customer in remote counselling	11	9	0.82	6	0.55
2. I can set goals together with the customer in remote counselling	11	10	0.91	10	0.91
3. I can form a confidential relationship with the customer in remote counselling	11	10	0.91	10	0.91
4. I can recognise the customer’s need for support and guidance in remote counselling	11	9	0.82	10	0.91
5. I can motivate the customer into action/self-care in remote counselling	11	9	0.82	10	0.91
6. I can take into consideration the special characteristics of online interaction (e.g. wording, addressing empathy) in remote counselling	10	7	0.7	7	0.7
7. I can recognise when the customer’s service (e.g. care or guidance) can be delivered remotely	11	9	0.82	9	0.82
8. I can guide the customer verbally in remote counselling	11	10	0.91	10	0.91
9. I can guide the customer by utilising a video connection in remote counselling	11	10	0.91	9	0.82
10. I can evaluate the customer’s situation (need for care or service) in remote counselling	11	8	0.73	9	0.82
11. I can guide the customer in writing (e.g. chat service) in remote counselling	11	7	0.64	7	0.7
12. I can evaluate whether customers receive equal service in remote counselling	11	8	0.73	7	0.7
13. I can recognise the customer‘s willingness to use digital solutions	11	10	0.91	9	0.82
14. I can act professionally in remote counselling	11	9	0.82	10	0.91
15. I can evaluate the customer’s digital readiness	11	10	0.91	9	0.82
16. I can guide the customer to find reliable information (e.g. from the Finnish Institute for Health and Welfare, Social Insurance Institution of Finland, Health Village, Health Library, Nursing Research Foundation)	11	5	0.45	7	0.64
Subscale S-CVI/Ave			0.80		0.80
Digital solutions as part of work					
17. The transfer to digital services is a positive change	12	12	1	11	0.92
18. Digital solutions should be used more in social and health services	12	12	1	12	1
19. I am motivated to use digital solutions in my work	12	12	1	12	1
20. I consider digital solutions as useful	12	12	1	11	0.92
21. I am interested in learning about digital solutions in my work	12	12	1	12	1
22. Digital solutions support my work	12	12	1	12	1
23. Digital services are a good way to deliver social and health services (e.g. customer work, care, rehabilitation)	12	10	0.83	11	0.92
24. Digital solutions are a natural part of my work	12	11	0.92	11	0.92
25. Digital solutions do not slow down my work	12	12	1	12	1
Subscale S-CVI/Ave			0.97		0.96
Information and communication technology (ICT) competence					
26. I can use the most common computer programs and services (e.g. email, intranet) in my work	12	12	1	11	0.92
27. I can use equipment based on information technology (e.g. computer) in my work	12	12	1	12	1
28. I can ask for help in information technology issues (e.g. ICT support)	12	11	0.92	11	0.92
29. I can use the patient/client information system in my work	12	12	1	12	1
30. I can solve most common information technology challenges (e.g. login problems, display settings, printer settings) in my work	12	10	0.83	12	1
Subscale S-CVI/Ave			0.95		0.97
Competence in utilising and evaluating digital solutions					
31. I can recognise what digital solutions are in social and health services	12	10	0.83	8	0.67
32. I can recognise factors (e.g. resources, motivation) that influence the utilisation of digital solutions	12	11	0.92	12	1
33. I can utilise digital solutions (e.g. smart devices, applications) in client care/guidance	12	11	0.92	12	1
34. I can utilise digital solutions creatively (e.g. usage according to different customer needs) in my work	12	10	0.83	10	0.83
35. I can boldly experiment and implement digital solutions in my work	12	10	0.83	10	0.83
36. I can explain digital social and health services (e.g. Health Villiage, Omaaolo) to clients	12	6	0.5	7	0.58
37. I can use my professional skills when using digital solutions.	12	8	0.6	9	0.75
38. I can critically evaluate new digital solutions.	12	11	0.92	11	0.92
Subscale S-CVI/Ave			0.79		0.82
Ethical competence related to digital solutions					
39. I can secure the customer’s privacy when using digital solutions.	11	9	0.82	10	0.91
40. I can ensure the secure processing of customer data	11	11	1	11	1
41. I can acknowledge the customer’s autonomy when using digital solutions	11	10	0.91	9	0.82
42. I can recognise the ethical aspects of digital solutions (e.g. freedom of choice, privacy, fairness)	11	10	0.91	9	0.82
Subscale S-CVI/Ave			0.91		0.89
S-CVI/Ave			0.86		0.87

*Recommendations*: The participants were concerned that this subscale does not apply to all categories of healthcare professionals, since they have different roles and scopes of practice, and not all healthcare professionals provide “counselling” (especially nurses). A further concern was about who the recipient of the counselling was. Participants recommended an extension of the definition of remote counselling to clarify the meaning of counselling and the recipient thereof. The definition was amended to “*digital (online, mobile, WhatsApp) counselling/patient guidance/health education/health literacy.”* Additionally, the subscale name was changed to align with the new definition of digital counselling. Further suggestions included replacing “act in reciprocal interaction” (item 1) with “engaging in mutually beneficial interaction” to enhance clarity (Supplementary Tables S1, S3) and adding contextually appropriate examples of sources of information for item 16 (Supplementary Table S1).

#### 3.2.3 Subscale 2: digital solutions as part of work

The S-CVI values for the subscale were acceptable (S-CVI_relevance_/Ave = 0.97; S-CVI_clarity_/Ave = 0.96; Table 1). The I-CVI_relevance_ were ≥0.83, with experts being unanimous on 7/9 items (I-CVI = 1; Table 1). Similarly, I-CVI_clarity_ were ≥0.92, with experts being unanimous on 5/9 items (I-CVI = 1; Table 1).

*Recommendations:* For items 17 and 20, it was recommended to add “in my work” to clarify the context. For item 23, it was recommended to replace “deliver” with “manage” as participants felt that information systems cannot “provide care” but can be used to improve management of social and health services. Additionally, it was recommended to remove “customer work” from the examples (Amended item: “*Digital services are a good way to manage social and health services*—e.g. *care, rehabilitation*”). For item 24 (Original item: “*Digital solutions are a natural part of my work*”) it was recommended that the word “natural” be removed to enhance the clarity of the item (Supplementary Table S1).

#### 3.2.4 Subscale 3: information and communication technology (ICT) competence

The S-CVI values for the subscale were acceptable (S-CVI_relevance_/Ave = 0.95; S-CVI_clarity_/Ave = 0.97; Table 1). I-CV I_relevance_ were ≥0.83, with experts being unanimous on 3/5 items (I-CVI = 1; Table 1). I-CV I_clarity_ were ≥0.92, with experts being unanimous on 3/5 items (I-CVI = 1; Table 1).

*Recommendations:* Participants recommended the removal of “in my work” from item 27 (Original item: “*I can use equipment based on information technology (*e.g.*, computer) in my work*”). The motivation was that someone may have the ability to use the equipment but may not have access to these functions at work as available technology differs in private and public healthcare contexts in South Africa. For item 30 (Original item: “*I can solve most common information technology challenges [*e.g. *login problems, display settings, printer settings] in my work*”), participants recommended changing the item to be more suitable to the South African context where information technology challenges such as login and printer issues are mostly handled by in-house IT support services due to how the systems are set up. The item was amended to refer to both the user’s ability to solve the problem and knowing how to ask for the necessary support (Amended item: “*I can solve or know how to ask for support with most common information technology challenges [*e.g. *login problems, display settings, printer settings] in my work*”; Supplementary Table S1).

#### 3.2.5 Subscale 4: competence in utilising and evaluating digital solutions

The S-CVI_relevance_/Ave = 0.79 was just below sufficient, while the S-CVI_clarity_/Ave = 0.82 was sufficient (Table 1). I-CV I_relevance_ were ≥0.83, except for items 36 (I-CVI = 0.5) and 37 (I-CVI = 0.60; Table 1). I-CV I_clarity_ were ≥0.83, except for items 31 (I-CVI = 0.67), 36 (I-CVI = 0.58), and 37 (I-CVI = 0.75; Table 1).

*Recommendations:* For item 36 it was recommended to replace the original examples of digital social and health services with examples that are applicable to the South African context. For item 37 “digital solutions” was replaced with “digital tools” (Supplementary Tables S1, S3). In terms of clarity, participants recommended that the meaning of “social and health services” in item 31 should be clarified, and the following explanation was added in collaboration with the original developers of the measure: “*all service levels from primary to specialized and public to private healthcare and welfare services*.” For items 36 and 37 the changes indicated for relevance also apply to clarity. Experts were unanimous on 2/8 items (I-CVI = 1; Supplementary Table S1).

#### 3.2.6 Subscale 5: ethical competence related to digital solutions

The S-CVI values for the subscale were acceptable (S-CVI_relevance_/Ave = 0.91; S-CVI_clarity_/Ave = 0.89; Table 1). I-CV I_relevance_ were ≥0.82, with experts being unanimous on item 40 (Table 1). I-CV I_clarity_ were ≥0.82, with experts being unanimous on item 40 (I-CVI = 1; Table 1).

*Recommendations*. For item 39 participants recommended that “secure” should be replaced with “ensure” (Amended item: “*I can ensure the client’s privacy when using digital tools”*). For item 41, participants indicated the need to clarify what is meant by the “client’s autonomy” and recommended adding the following explanation: “*right to make their own choices without coercion*.” For item 42. adding more examples of ethical aspects that should be considered when using digital tools was recommended (Supplementary Table S1).

### 3.3 *DigiComInf* content validity ratings for relevance and clarity and participant recommendations

#### 3.3.1 Overall ratings for the *DigiComInf*

The S-CVI values for the total measure were acceptable (S-CVI_relevance_/Ave = 0.90; S-CVI_clarity_/Ave = 0.89; Table 2). Changes across the measure include changing “digital solutions” to “digital tools” to ensure conceptual clarity and adding a definition for “digital competence” in the administration instructions. In collaboration with the original authors, the following definition was added “*In healthcare, digital competence includes sufficient knowledge and skills in the use of digital technologies needed to provide high quality ethical patient care, social and communication skills to apply digital technologies into health care delivery and the willingness of healthcare professionals to integrate digitalization in their professional context*.” In addition, we added a “not applicable” option to the response scale and amended the administration instructions accordingly. Supplementary Table S3 shows the comparison between the original and amended items of *DigicomInf*, and Supplementary Table S4 shows the final version of *DigiComInf* suggested for use in South Africa.

**Table 2:** Ratings and CVI values for DigiComInf.

Item	# Experts	Relevance		Clarity	
		R3/R4	I-CVI	C3/C4	I-CVI
Support from management					
1. My manager’s example supports the development of my digital competence	12	12	1	11	0.92
2. My manager supports the implementation of digital solutions	12	12	1	12	1
3. My manager gives feedback about the development of my digital competence	12	12	1	11	0.92
4. My manager can lead the development of my digital competence (e.g. prediction of competence development, communication, clear guidance, support for renewal and participation)	11	7	0.64	8	0.73
5. My manager supports my participation in continuing education to strengthen my digital competence	12	12	1	12	1
6. Top management supports the uptake of digital solutions	12	12	1	11	0.92
Subscale S-CVI/Ave			0.94		0.92
Organisational practices as part of digital competence development					
7. Education about the digital solutions used at my work has been sufficient	12	11	0.92	10	0.83
8. Digital competence development is planned in my unit according to individual needs	12	11	0.92	11	0.92
9. The orientation for digital solutions is conducted systematically at my work unit	12	11	0.92	10	0.83
10. My organisation’s practices support opportunities to develop my digital competence	12	12	1	10	0.83
Subscale S-CVI/Ave			0.94		0.85
Colleagues’ adoption and influence					
11. Colleagues are not reluctant to start using digital solutions at work	12	12	1	10	0.83
12. The implementation of digital solutions has been perceived positively in my work community	12	12	1	12	1
13. Colleagues are eager to develop their own work on digital solutions	12	11	0.92	10	0.83
14. Colleagues do not have a negative influence on the development of my digital competence	12	12	1	11	0.92
15. Colleagues in my work community have mainly a good level of digital competence	11	11	0.92	9	0.82
Subscale S-CVI/Ave			0.97		0.88
S-CVI/Ave			0.95		0.89

#### 3.3.2 Subscale 1: support from management

The S-CVI values were acceptable (S-CVI_relevance_/Ave = 0.94; S-CVI_clarity_/Ave = 0.92; Table 2).

Participants were unanimous on all items (I-CVI = 1) in terms of relevance, except for item 4 with a I-CVI value (0.64) below the ≥0.78 cut-off (Table 2). I-CVI_clarity_ were ≥0.92, except for item 4 (I-CVI = 0.73), with experts being unanimous on items 2 and 5 (I-CVI = 1; Table 2).

*Recommendations:* For items 1–5 the participants recommended that the level of management be clarified as “line manager.” Additionally, item 1 was recommended to be amended to refer to the “line manager’s vision” rather than the “manger’s example” that support the development of digital competence (Supplementary Table S3).

#### 3.3.3 Subscale 2: Organisational practices as part of digital competence development

The S-CVI values were acceptable (S-CVI_relevance_/Ave = 0.94; S-CVI_clarity_/Ave = 0.86; Table 2). I-CV I_relevance_ were ≥0.92, with participants unanimous on item 10 (I-CVI = 1; Table 2). I-CVI_clarity_ were ≥ 0.83 (Table 2).

*Recommendations:* It was recommended amending item 10 to refer to the “organisation” rather than the “organisation’s practices” that support the development of digital competence (Supplementary Table S3).

#### 3.3.4 Subscale 3: colleagues’ adoption and influence

The S-CVI values were acceptable (S-CVI_relevance_/Ave = 0.97; S-CVI_clarity_/Ave = 0.88; Table 2). I-CV I_relevance_ were ≥0.92, with participants unanimous on items 11, 12, and 14 (I-CVI = 1; Table 2). I-CVI_clarity_ were ≥0.83, with participants unanimous about item 12 (Table 2).

*Recommendations:* Item 13 was adapted to be more contextually relevant. The original item (“*Colleagues are eager to develop their own work on digital solutions*”) was amended to “*Colleagues are eager to develop the skills to use digital tools in their own work*,” as individuals are not necessarily able to develop their own work on digital solutions (Supplementary Table S3).

## 4 Discussion

### 4.1 Overall content validity of the measures

The S-CVI_relevance_ /Ave and S-CVI_clarity_/Ave values for the total measures and their subscales were sufficient, except for the S-CVI_relevance_/Ave value of the *“Competence in utilising and evaluating digital solutions*” subscale (*DigiHealthCom*) which was just below the ≥0.80 cut-off (S-CVI/Ave = 0.79). I-CVI values were mostly ≥0.78 except for a few exceptions that occurred mainly in the “*Human-centred remote counselling competence*” subscale (*DigiHealthCom*). For *DigiComInf*, only item 4 (“*My manager can lead the development of my digital competence [*e.g. *prediction of competence development, communication, clear guidance, support for renewal and participation”]*) had I-CVI_relevance_ and I_CVI_clarity_ values below the ≥0.78 cut-off. Missing expert ratings could influence content validity. For *DigiHealthCom* most items were rated by 11 of the 12 experts, while only two items of *DigiComInf* were not rated by all experts (rated by 11/12 experts). The effect of missing ratings may therefore be less impactful for *DigiComInf*. Although the study had final ratings by between 10 and 12 reviewers for which I-CVI values ≥78 is considered acceptable (12), the findings may need to be interpreted cautiously. Missing ratings for the “*Human-centred remote counselling competence*” subscale (*DigiHealthCom*) may be due to the general concern among participants that the subscale does not apply to all healthcare professionals as not all healthcare professionals provide counselling. The amendments to *DigiHealthCom* and *DigiComInf* involved some complex and some simple adaptations to be suitable to the South African context. Content validity values were mostly acceptable with some suggested amendments. More complex amendments included defining terms to highlight their meaning and to clarify the context. Simpler amendments included changing wording to be suitable to the context.

### 4.2 Conceptual clarity and contextual relevance

Concerns across the measures related to two main categories, namely conceptual clarity and contextual relevance. Most concerns related to *DigiHealthCom* and centred around conceptual clarity and the relevance of the “*human-centred remote counselling competence*” subscale to different healthcare professionals. In South Africa, counselling is viewed as a core function for psychologists and mental health nurses, while counselling in general nursing refers to health promotion advice and support (16, 17). It was also unclear who the recipients of the counselling are. To address these concerns, an appropriate, context-relevant definition for “digital counselling” was added, which clarified the application of the subscale to different healthcare professionals, as well as who the recipients of the counselling are. Additionally, a “not applicable” option was added to the response scale. Participants also suggested that a definition must be added for “digital competence” (*DigiComInf*) to clarify the construct, and a context-relevant definition was added to the administration instructions. Further concerns regarding contextual relevance were addressed by replacing words with suitable alternatives to cater for differing literacy levels (items 1, 41 *DigiHealthCom*); adapting items to provide contextually relevant examples of sources of information or available services (items 16, 36 *DigiHealthCom*); and clarifying and adapting items (item 17, 20, 23, 24, 27, 30, 31, 42 *DigiHealhCom*) to fit the South African context.

### 4.3 The importance of user input in content validity

The importance of exploring the content validity of a measure qualitatively through user input is illustrated in Connell et al. (18). In the Connell study, interviews and focus groups were used to explore the type of items that users (as opposed to experts) felt were important for inclusion in the *Recovering Quality of Life* measure for people with mental health difficulties. Service users in the Connell study raised several concerns that some items (a) were not relevant to their mental health or quality of life (e.g., “feeling cared for” was considered more important for quality of live than “feeling loved”); (b) were vague or too abstract and could be particularly difficult to answer if a test-taker was distressed or if answering the item would elicit too much mental effort; (c) were judgemental in what was considered to be a good outcome (e.g., items related to helping someone could cause feelings of guilt if a person was unable to help); (d) were ambiguous, and (e) others were sensitive and could cause distress to the test-taker [e.g., the directness of how questions related to suicide were asked; (18)]. This study also highlighted the importance of considering the developmental phase of the test-taker as young adults felt that confusion about ‘who I am’ was normal for their age group and not necessarily a sign of poor mental health (18). Though not specifically identified in this study, the possible effect of test-taker behaviour on the psychometric properties should be considered. Item-formulation can influence test-taker experience [e.g., items that are perceived too difficult, or too long require more cognitive effort; (19)] and/or elicit emotional responses (e.g., feeling upset or guilty) which may influence how test-takers respond to items (18). In Connell et al. (18) service users indicated that they would not answer truthfully, or not at all, if the items they perceived to be problematic were included in the measure. Such test-taker behaviour could have unfavourable consequences psychometrically, threatening validity and reliability (20, 21).

It is also important to determine content validity when measures have been translated or when the same language version will be used in another context. The *DigiHealthCom* and *DigiComInfl* were originally developed in Finnish and translated into English for the United Kingdom context (8). These measures were evaluated for linguistic, cultural, professional, and organisational fit for use in the UK context, and required only minor linguistic adaptations into British English to be suitable to the context (5). However, these translations (UK English) of the measures required some contextual and linguistic changes to be suitable to South Africa and its specific diverse settings. Some of these changes related specifically to the availability of, and access to, technology in private vs. public healthcare settings (e.g., item 27, *DigiHealthCom*), or the internal practices and structures (e.g., item 30, *DigiHealthCom*, items 1–6 *DigiComInf*) that is more typical of South African healthcare settings. This illustrate that even if a measure is available in the desired language, it must still be content validated to accommodate contextual differences.

In addition, when measures are translated, the meaning of items may get lost in translation, especially when there is not an exact translation for a specific word and an equivalent word is used for translation (22–24). Such instances raise questions, such as whether the item or items in question are still representative of the target construct, and whether it is still the target construct or perhaps a related construct that is measured. In this regard, Larson (23) indicated that when the contextual meaning of a construct is not considered, the very concept that was supposed to be measured may be lost.

### 4.4 Future steps

As part of the rollout of the instrument in the South African context, additional psychometric testing will be conducted. This includes determining the factorial validity, measurement invariance, and reliability of the scales once the data collection is completed.

## 5 Limitations

While it is essential to determine the content validity of measures, there are limitations to this study. Firstly, item ratings are inherently subjective and based on whether the expert experienced the item as relevant or clear to their context. Secondly, although the participants are experts in their respective fields and from diverse nursing contexts, differing levels of experience and varying experiences in diverse professional contexts may have influenced the interpretation of items and therefore the ratings. Thirdly, although participants completed the content validity rating scale independently and prior to discussion in the focus group, consensus discussion may have influenced the ratings of participants during the focus group. Fourthly, missing expert ratings may have influenced content validity, hence the ratings may need to be interpreted cautiously. Fifthly, since the sample consisted primarily of nurses—with a limited number of participants from other healthcare professions—the measures may not be applicable to other healthcare professionals beyond the nursing profession and should be investigated for other health professions. Lastly, although small samples can be used for content validity, the generalisability of the results to the larger nursing population may be limited.

## 6 Recommendations

Determining the content validity of *DigiHealthCom* and *DigiComInf* constitutes only the initial step towards determining the psychometric soundness of these measures for the nursing profession in South Africa. Future research should explore the factorial validity, construct validity, predictive validity, test–retest stability, measurement invariance, and reliability of *DigiHealthCom* and *DigiComInf* for the nursing and other healthcare professions in South Africa.

## 7 Conclusion

This study constitutes initial evidence for the content validity of *DigiHealthCom* and *DigiComInf* among nurses in the South African context, with some adaptation required to be suitable to the South African context. Further research should further validate these measures among nurses and other healthcare professions in South Africa.

## Data Availability

The raw data supporting the conclusions of this article will be made available by the authors on request, without undue reservation.

## References

[1] Laurenza E Quintano M Schiavone F Vrontis D. The effect of digital technologies adoption in healthcare industry: a case based analysis. BPMJ. (2018) 24:1124–44. doi: 10.1108/BPMJ-04-2017-0084

[2] Wendel J Hofmann A-L Widmann J Wöckel A Heuschmann P Reese J-P. Perceived benefits and disadvantages for healthcare professionals when implementing digital health technologies in breast cancer care: a systematic review. DIGITAL HEALTH. (2025) 11:20552076251404497. doi: 10.1177/20552076251404497, 41362304 PMC12681584

[3] Ilomäki L Paavola S Lakkala M Kantosalo A. Digital competence – an emergent boundary concept for policy and educational research. Educ Inf Technol. (2016) 21:655–79. doi: 10.1007/s10639-014-9346-4

[4] Jarva E Mikkonen K Andersson J Tuomikoski A-M Kääriäinen M Meriläinen M . Aspects associated with health care professionals’ digital health competence development – a qualitative study. FinJeHeW. (2022) 14:79–91. doi: 10.23996/fjhw.111771

[5] Erfani G McCREADY J Gibson B Nichol B Unsworth J Jarva E . Factors influencing digital health competence among healthcare professionals: a cross-sectional study. Appl Nurs Res. (2025) 82:151922. doi: 10.1016/j.apnr.2025.151922, 40086941

[6] Le Roux L Bimerew M Chipps J-A. A survey of nurse informatics competencies of professional nurses in clinical practice public hospitals in South Africa. Int. J. Afr. Nurs. Sci. (2024) 21. doi: 10.1016/j.ijans.2024.100783 39049329

[7] Le Roux L Bimerew M Chipps J. Preparation of nursing students in nursing informatics competencies for the South African healthcare practice environment. BMC Nurs. (2024) 23:923. doi: 10.1186/s12912-024-02614-4, 39696363 PMC11657814

[8] Jarva E Oikarinen A Andersson J Tomietto M Kääriäinen M Mikkonen K. Healthcare professionals’ digital health competence and its core factors; development and psychometric testing of two instruments. Int J Med Inform. (2023) 171:104995. doi: 10.1016/j.ijmedinf.2023.104995, 36689840

[9] Cook DA Beckman TJ. Current concepts in validity and reliability for psychometric instruments: theory and application. Am J Med. (2006) 119:166.e7–166.e16. doi: 10.1016/j.amjmed.2005.10.036, 16443422

[10] Polit DF Beck CT. The content validity index: are you sure you know what’s being reported? Critique and recommendations. Res Nurs Health. (2006) 29:489–97. doi: 10.1002/nur.20147, 16977646

[11] Haynes SN Richard DCS Kubany ES. Content validity in psychological assessment: a functional approach to concepts and methods. Psychol Assess. (1995) 7:238–47. doi: 10.1037/1040-3590.7.3.238

[12] Lynn MR. Determination and quantification of content validity. Nurs Res. (1986) 35:382–6. doi: 10.1097/00006199-198611000-00017 3640358

[13] Polit DF Beck CT Owen SV. Is the CVI an acceptable indicator of content validity? Appraisal and recommendations. Res Nurs Health. (2007) 30:459–67. doi: 10.1002/nur.20199, 17654487

[14] Waltz CF Strickland OL Lenz ER. Measurement in Nursing and Health Research. 3rd ed. New York: Springer (2005).

[15] Davis LL. Instrument review: getting the most from a panel of experts. Appl Nurs Res. (1992) 5:194–7. doi: 10.1016/S0897-1897(05)80008-4

[16] Amendment No R704. (n.d.). Regulations Defining the Scope of the Profession of Psychology. Health Professions Act, 1974 (Act No. 56 of 1974).

[17] Mental Health Care Act 17 of 2002. (n.d.). Government Gazette No 24024, 6 November 2022.

[18] Connell J Carlton J Grundy A Taylor Buck E Keetharuth AD Ricketts T . The importance of content and face validity in instrument development: lessons learnt from service users when developing the recovering quality of life measure (ReQoL). Qual Life Res. (2018) 27:1893–902. doi: 10.1007/s11136-018-1847-y, 29675691 PMC5997715

[19] Csányi R Molnár G. Item- and person-level factors in test-taking disengagement: multilevel modelling in a low-stakes context. Int. J. Educ. Res. Open. (2024) 7:100373. doi: 10.1016/j.ijedro.2024.100373

[20] Akinboboye JT Ayanwale MA Adewuni DA Vincent YI. Impact of unanswered questions on examinees’ latent traits: an item response theory perspective. Afr J Psychol Assess. (2024) 6:a161. doi: 10.4102/AJOPA.v6i0.161, 40406657 PMC12082205

[21] Kreitchmann RS Abad FJ Ponsoda V Nieto MD Morillo D. Controlling for response biases in self-report scales: forced-choice vs. psychometric modeling of Likert items. Front Psychol. (2019) 10:2309. doi: 10.3389/fpsyg.2019.02309, 31681103 PMC6803422

[22] Cromhout A Schutte L Wissing MP Wilson Fadiji A Guse T Mbowa S. Psychometric properties of the harmony in life scale in south African and Ghanaian samples. AJOPA. (2023) 5:a122. doi: 10.4102/ajopa.v5i0.122, 40406205 PMC12082207

[23] Larson. Lost in Translation: Implications for Translating Psychometric Tests as seen with the Nunchi Scale. [Masters thesis, Brigham Young University]. (2022). Available online at: https://scholarsarchive.byu.edu/cgi/viewcontent.cgi?article=10696&context=etd

[24] Lomas T. Experiential cartography and the significance of “untranslatable” words. Theory Psychol. (2018) 28:476–95. doi: 10.1177/0959354318772914

[25] Yusoff MSB. ABC of content validation and content validity index calculation. Educ. Med. J. (2019) 11:49–54. doi: 10.21315/eimj2019.11.2.6

