# Supplementary material for: Content validity of digital health competence measures for nursing in South Africa
Source: Front Public Health. 2026 Sep 7;14:1874126. doi: 10.3389/fpubh.2026.1874126 (PMC13590713; doi:10.3389/fpubh.2026.1874126)
Supplement: Supplementary file 1 [file Supplementary_file_1.pdf]

Supplementary Material

1 Supplementary Table S1

Comparison of Original and Amended items of DigiHealthCom

Original	Amended
Based on the following statements, assess your general level of competence in using digital solutions, where: 1 = completely disagree; 2 = disagree; 3 = agree; 4 = completely agree.	Based on the following statements, assess your general level of competence in using digital *tools*, where: *0 = not applicable*; 1 = completely disagree; 2 = disagree; 3 = agree; 4 = completely agree.
**Human-centred remote counselling competence**	**Human-centred *digital (online, mobile, WhatsApp)* counselling/*patient guidance/health education/health literacy.***
1. I can act in reciprocal (aiming towards respect and equality) interaction with the customer in remote counselling	1. I can *engage in mutually beneficial* interaction (aiming towards respect and equality) with the *client* in *digital (online, mobile, WhatsApp) counselling/patient guidance/health education/health literacy.*
2. I can set goals together with the customer in remote counselling	2. I can set goals together with the *client* in *digital (online, mobile, WhatsApp)* counselling/*patient guidance/health education/health literacy*
3. I can form a confidential relationship with the customer in remote counselling	3. I can form a confidential relationship with the *client* in *digital (online, mobile, WhatsApp)* counselling/*patient guidance/health education/health literacy.*
4. I can recognise the customer's need for support and guidance in remote counselling	4. I can recognise the *client's* need for support and guidance in *digital (online, mobile, WhatsApp)* counselling/*patient guidance/health education/health literacy.*
5. I can motivate the customer into action/self-care in remote counselling	5. I can motivate the *client* into action/self-care in *digital (online, mobile, WhatsApp)* counselling/*patient guidance/health education/health literacy.*
6. I can take into consideration the special characteristics of online interaction (e.g. wording, addressing empathy) in remote counselling	6. I can take into consideration the special characteristics of online interaction (e.g. wording, addressing empathy) in *digital (online, mobile, WhatsApp)* counselling/*patient guidance/health education/health literacy.*
7. I can recognise when the customer's service (e.g. care or guidance) can be delivered remotely	7. I can recognise when the *client's* service (e.g. care or guidance) can be delivered *digitally (online, mobile, WhatsApp).*
8. I can guide the customer verbally in remote counselling	8. I can guide the *client* verbally in *digital (online, mobile, WhatsApp)* counselling/*patient guidance/health education/health literacy.*

9. I can guide the customer by utilising a video connection in remote counselling	9. I can guide the *client* by utilising a video connection in *digital (online, mobile, WhatsApp)* counselling/*patient guidance/health education/health literacy.*
10. I can evaluate the customer's situation (need for care or service) in remote counselling	10. I can evaluate the *client's* situation (need for care or service) in *digital (online, mobile, WhatsApp)* counselling/*patient guidance/health education/health literacy.*
11. I can guide the customer in writing (e.g. chat service) in remote counselling	11. I can guide the *client* in writing (e.g. chat service) in *digital (online, mobile, WhatsApp)* counselling/*patient guidance/health education/health literacy.*
12. I can evaluate whether customers receive equal service in remote counselling	12. I can evaluate whether *clients* receive equal service in *digital (online, mobile, WhatsApp)* counselling/*patient guidance/health education/health literacy.*
13. I can recognise the customer‘s willingness to use digital solutions	13. I can recognise the *client's* willingness to use *digital (online, mobile, WhatsApp)* counselling/*patient guidance/health education/health literacy*.
14. I can act professionally in remote counselling	14. I can act professionally in *digital (online, mobile, WhatsApp)* counselling/*patient guidance/health education/health literacy.*
15. I can evaluate the customer's digital readiness	15. I can evaluate the *client's* digital readiness
16. I can guide the customer to find reliable information (e.g., from the Finnish Institute for Health and Welfare, Social Insurance Institution of Finland, Health Village, Health Library, Nursing Research Foundation)	16. I can guide the *client* to find reliable information (*e.g., from the National Department of Health, Provincial Health Department Websites, National Institute for Communicable Diseases, Department of Social Development, SASSA*)
**Digital solutions as part of work**	**Digital *tools* as part of work**
17. The transfer to digital services is a positive change	17. The transfer to digital services is a positive change *in my work*
18. Digital solutions should be used more in social and health services	18. Digital *tools* should be used more in social and health services
19. I am motivated to use digital solutions in my work	19. I am motivated to use digital *tools* in my work
20. I consider digital solutions as useful	20. I consider digital *tools* as useful *in my work*
21. I am interested in learning about digital solutions in my work	21. I am interested in learning about digital *tools* in my work
22. Digital solutions support my work	22. Digital *tools* support my work
23. Digital services are a good way to deliver social and health services (e.g. customer work, care, rehabilitation	23. Digital services are a good way to *manage* social and health services (e.g. ~~customer work~~, care, rehabilitation)
24. Digital solutions are a natural part of my work	24. Digital *tools* are ~~a natural~~ part of my work

25. Digital solutions do not slow down my work	25. Digital *tools* do not slow down my work
**Information and communication technology (ICT) competence**	**Information and communication technology (ICT) competence**
26. I can use the most common computer programs and services (e.g. email, intranet) in my work	26. I can use the most common computer programs and services (e.g. email, intranet) in my work
27. I can use equipment based on information technology (e.g. computer) in my work	27. I can use equipment based on information technology (e.g. computer) ~~in my work~~
28. I can ask for help in information technology issues (e.g. ICT support)	28. I can ask for help in information technology issues (e.g. ICT support)
29. I can use the patient/client information system in my work	29. I can use the patient/client information system in my work
30. I can solve most common information technology challenges (e.g. login problems, display settings, printer settings) in my work	30. I *can solve or know how to ask for support* with most common information technology challenges (e.g. login problems, display settings, printer settings) in my work
**Competence in utilising and evaluating digital solutions**	**Competence in utilising and evaluating digital *tools***
31. I can recognise what digital solutions are in social and health services	31. I can recognise what digital *tools* are in social and health services (*all service levels from primary to specialized and public to private healthcare and welfare services*)
32. I can recognise factors (e.g. resources, motivation) that influence the utilisation of digital solutions	32. I can recognise factors (e.g. resources, motivation) that influence the utilisation of digital *tools*
33. I can utilise digital solutions (e.g. smart devices, applications) in customer care/guidance	33. I can utilise digital *tools* (e.g. smart devices, applications) in *client* care/guidance
34. I can utilise digital solutions creatively (e.g. usage according to different customer needs) in my work	34. I can utilise digital *tools* creatively (e.g. usage according to different *client* needs) in my work
35. I can boldly experiment and implement digital solutions in my work	35. I can boldly experiment and implement digital *tools* in my work
36. I can explain digital social and health services (e.g. Health Villiage, Omaaolo) to customers	36. I can explain digital social and health services (*e.g. National Department of Health, Provincial Health Department Websites, National Institute for Communicable Diseases, Department of Social Development, SASSA, etc*) to *clients*
37. I can use my professional skills when using digital solutions.	37. I can use my professional skills when using digital *tools*
38. I can critically evaluate new digital solutions.	38. I can critically evaluate new digital *tools*
**Ethical competence related to digital solutions**	**Ethical competence related to digital *tools***
39. I can secure the customer's privacy when using digital solutions.	39. I can *ensure* the *client's* privacy when using digital *tools*

40. I can ensure the secure processing of customer data	40. I can ensure the secure processing of *client* data
41. I can acknowledge the customer's autonomy when using digital solutions	41. I can acknowledge the *client's* autonomy *(right to make their own choices without coercion*) when using digital *tools*
42. I can recognise the ethical aspects of digital solutions (e.g. freedom of choice, privacy, fairness)	42. I can recognise the ethical aspects of *using* digital *tools* (e.g. freedom of choice, privacy, fairness, *patient information, client data, confidentiality, etc.*)

2 Supplementary Table S2

Recommended Version of DigiHealthCom for Use in South Africa

**Based on the following statements, assess your general level of competence in using digital tools, where: 0 = not applicable; 1 = completely disagree; 2 = disagree; 3 = agree; 4 = completely agree.**					
	**n/a 0**	**CD 1**	**D 2**	**A 3**	**CA 4**
**Human-centred digital (online, mobile, WhatsApp) counselling/patient guidance/health education/health literacy.**					
1. I can engage in mutually beneficial interaction (aiming towards respect and equality) with the client in digital (online, mobile, WhatsApp) counselling/patient guidance/health education/health literacy.	0	1	2	3	4
2. I can set goals together with the client in digital (online, mobile, WhatsApp) counselling/patient guidance/health education/health literacy.	0	1	2	3	4
3. I can form a confidential relationship with the client in digital (online, mobile, WhatsApp) counselling/patient guidance/health education/health literacy.	0	1	2	3	4
4. I can recognise the client's need for support and guidance in digital (online, mobile, WhatsApp) counselling/patient guidance/health education/health literacy.	0	1	2	3	4
5. I can motivate the client into action/self-care in digital (online, mobile, WhatsApp) counselling/patient guidance/health education/health literacy.	0	1	2	3	4
6. I can take into consideration the special characteristics of online interaction (e.g. wording, addressing empathy) in digital (online, mobile, WhatsApp) counselling/patient guidance/health education/health literacy.	0	1	2	3	4
7. I can recognise when the client's service (e.g. care or guidance) can be delivered digitally (online, mobile, WhatsApp).	0	1	2	3	4
8. I can guide the client verbally in digital (online, mobile, WhatsApp) counselling/patient guidance/health education/health literacy.	0	1	2	3	4
9. I can guide the client by utilising a video connection in digital (online, mobile, WhatsApp) counselling/patient guidance/health education/health literacy.	0	1	2	3	4
10. I can evaluate the client's situation (need for care or service) in digital (online, mobile, WhatsApp) counselling/patient guidance/health education/health literacy.	0	1	2	3	4
11. I can guide the client in writing (e.g. chat service) in digital (online, mobile, WhatsApp) counselling/patient guidance/health education/health literacy.	0	1	2	3	4
12. I can evaluate whether clients receive equal service in digital (online, mobile, WhatsApp) counselling/patient guidance/health education/health literacy.	0	1	2	3	4
13. I can recognise the client's willingness to use digital (online, mobile, WhatsApp) counselling/patient guidance/health education/health literacy.	0	1	2	3	4

14. I can act professionally in digital (online, mobile, WhatsApp) counselling/patient guidance/health education/health literacy.	0	1	2	3	4
15. I can evaluate the client's digital readiness	0	1	2	3	4
16. I can guide the client to find reliable information (e.g., from the National Department of Health, Provincial Health Department Websites, National Institute for Communicable Diseases, Department of Social Development, SASSA, etc.)	0	1	2	3	4
**Digital tools as part of work**					
17. The transfer to digital services is a positive change in my work	0	1	2	3	4
18. Digital tools should be used more in social and health services	0	1	2	3	4
19. I am motivated to use digital tools in my work	0	1	2	3	4
20. I consider digital tools as useful in my work	0	1	2	3	4
21. I am interested in learning about digital tools in my work	0	1	2	3	4
22. Digital tools support my work	0	1	2	3	4
23. Digital services are a good way to manage social and health services (e.g. care, rehabilitation)	0	1	2	3	4
24. Digital tools are part of my work	0	1	2	3	4
25. Digital solutions do not slow down my work	0	1	2	3	4
**Information and communication technology (ICT) competence**					
26. I can use the most common computer programs and services (e.g. email, intranet) in my work	0	1	2	3	4
27. I can use equipment based on information technology (e.g. computer)	0	1	2	3	4
28. I can ask for help in information technology issues (e.g. ICT support)	0	1	2	3	4
29. I can use the patient/client information system in my work	0	1	2	3	4
30. I can solve most common information technology challenges (e.g. login problems, display settings, printer settings) in my work	0	1	2	3	4
**Competence in utilising and evaluating digital tools**					
31. I can recognise what digital tools are in social and health services (all service levels from primary to specialized and public to private healthcare and welfare services)	0	1	2	3	4
32. I can recognise factors (e.g. resources, motivation) that influence the utilisation of digital tools	0	1	2	3	4
33. I can utilise digital tools (e.g. smart devices, applications) in client care/guidance	0	1	2	3	4

34. I can utilise digital tools creatively (e.g. usage according to different client needs) in my work	0	1	2	3	4
35. I can boldly experiment and implement digital tools in my work	0	1	2	3	4
36. I can explain digital social and health services (e.g. National Department of Health, Provincial Health Department Websites, National Institute for Communicable Diseases, Department of Social Development, SASSA, etc) to clients	0	1	2	3	4
37. I can use my professional skills when using digital tools	0	1	2	3	4
38. I can critically evaluate new digital tools	0	1	2	3	4
**Ethical competence related to digital tools**					
39. I can ensure the client's privacy when using digital tools	0	1	2	3	4
40. I can ensure the secure processing of client data	0	1	2	3	4
41. I can acknowledge the client's autonomy (right to make their own choices without coercion) when using digital tools	0	1	2	3	4
42. I can recognise the ethical aspects of using digital tools (e.g. freedom of choice, privacy, fairness, patient information, client data, confidentiality, etc.)	0	1	2	3	4

3 Supplementary Table S3

Comparison of Original and Amended items of DigiComInf

**Original**	**Amended**
Assess the level of agreement to the following statements on the aspects associated with digital competence, where: 1 = completely disagree; 2 = disagree; 3 = agree; 4 = completely agree.	Assess the level of agreement to the following statements on the aspects associated with digital competence, where: *0 = not applicable;* 1 = completely disagree; 2 = disagree; 3 = agree; 4 = completely agree.<br><br>*In healthcare, digital competence includes sufficient knowledge and skills in the use of digital technologies needed to provide high quality ethical patient care, social and communication skills to apply digital technologies into health care delivery and the willingness of healthcare professionals to integrate digitalization in their professional context.*
**Support from management**	**DigiComInf - Support from management**
1. My manager's example supports the development of my digital competence	1. My *line* manager's *vision* supports the development of my digital competence
2. My manager supports the implementation of digital solutions	2. My *line* manager supports the implementation of digital *tools*
3. My manager gives feedback about the development of my digital competence	3. My *line* manager gives feedback about the development of my digital competence
4. My manager can lead the development of my digital competence (e.g. prediction of competence development, communication, clear guidance, support for renewal and participation)	4. My *line* manager can lead the development of my digital competence (e.g. prediction of competence development, communication, clear guidance, support for renewal and participation)
5. My manager supports my participation in continuing education to strengthen my digital competence	5. My *line* manager supports my participation in continuing education to strengthen my digital competence
6. Top management supports the uptake of digital solutions	6. Top management supports the uptake of digital *tools*
**Organisational practices as part of digital competence development**	**Organisational practices as part of digital competence development**
7. Education about the digital solutions used at my work has been sufficient	7. Education about the digital *tools* used at my work has been sufficient
8. Digital competence development is planned in my unit according to individual needs	8. Digital competence development is planned in my unit according to individual needs
9. The orientation for digital solutions is conducted systematically at my work unit	9. The orientation for digital *tools* is conducted systematically at my work unit
10. My organisation's practices support opportunities to develop my digital competence	10. My *organisation*~~'s~~ ~~*practices*~~ *support* opportunities to develop my digital competence
**Colleagues' adoption and influence**	**Colleagues' adoption and influence**
11. Colleagues are not reluctant to start using digital solutions at work	11. Colleagues are not reluctant to start using digital *tools* at work

12. The implementation of digital solutions has been perceived positively in my work community	12. The implementation of digital *tools* has been perceived positively in my work community
13. Colleagues are eager to develop their own work on digital solutions	13. Colleagues are eager to develop *the skills to use digital tools in* their own work ~~on digital solutions.~~
14. Colleagues do not have a negative influence on the development of my digital competence	14. Colleagues do not have a negative influence on the development of my digital competence
15. Colleagues in my work community have mainly a good level of digital competence	15. Colleagues in my work community have mainly a good level of digital competence

4 Supplementary Table S4

Recommended Version of DigiComInf for Use in South Africa

Assess the level of agreement to the following statements on the aspects associated with digital competence, where: 0 = not applicable; 1 = completely disagree; 2 = disagree; 3 = agree; 4 = completely agree.

In healthcare, digital competence includes sufficient knowledge and skills in the use of digital technologies needed to provide high quality ethical patient care, social and communication skills to apply digital technologies into health care delivery and the willingness of healthcare professionals to integrate digitalization in their professional context.

	**n/a**<br>**0**	**CD**<br>**1**	**D**<br>**2**	**A**<br>**3**	**CA**<br>**4**
**Support from management**					
1. My line manager's vision supports the development of my digital competence	0	1	2	3	4
2. My line manager supports the implementation of digital tools	0	1	2	3	4
3. My line manager gives feedback about the development of my digital competence	0	1	2	3	4
4. My line manager can lead the development of my digital competence (e.g. prediction of competence development, communication, clear guidance, support for renewal and participation)	0	1	2	3	4
5. My line manager supports my participation in continuing education to strengthen my digital competence	0	1	2	3	4
6. Top management supports the uptake of digital tools	0	1	2	3	4
**Organisational practices as part of digital competence development**					
7. Education about the digital tools used at my work has been sufficient	0	1	2	3	4
8. Digital competence development is planned in my unit according to individual needs	0	1	2	3	4
9. The orientation for digital tools is conducted systematically at my work unit	0	1	2	3	4
10. My organisation support opportunities to develop my digital competence	0	1	2	3	4
**Colleagues' adoption and influence**					
11. Colleagues are not reluctant to start using digital tools at work	0	1	2	3	4
12. The implementation of digital tools has been perceived positively in my work community	0	1	2	3	4
13. Colleagues are eager to develop the skills to use digital tools in their own work	0	1	2	3	4
14. Colleagues do not have a negative influence on the development of my digital competence	0	1	2	3	4
15. Colleagues in my work community have mainly a good level of digital competence	0	1	2	3	4
